# Comparison of Analgesic Prescriptions for Dental Pain and Patient Pain Outcomes Before vs After an Opioid Reduction Initiative

**DOI:** 10.1001/jamanetworkopen.2022.27219

**Published:** 2022-08-17

**Authors:** Qirong Huang, Linda Rasubala, Richard H. Gracely, Junad Khan, Eli Eliav, Yanfang Ren

**Affiliations:** 1 Eastman Institute for Oral Health, University of Rochester Medical Center, Rochester, New York

## Abstract

This cross-sectional study compares prescribing patterns of opioid and nonopioid analgesics and patients’ dental pain outcomes before vs after implementation of an opioid reduction initiative at a single dental clinic.

## Introduction

Dentists frequently prescribe opioids for dental pain and contribute substantially to new and persistent opioid use.^1^ Although the American Dental Association recommends nonsteroidal antiinflammatory drugs (NSAIDs) for managing pain,^2^ opioids continue to be used more than nonopioids.^3^ This may partly be explained by the lack of alternatives to opioids, especially when NSAIDs or acetaminophen are contraindicated or ineffective.^4^ We hypothesized that a multimodal analgesia strategy with NSAIDs, acetaminophen, and gabapentin would be associated with minimized use of opioids for dental pain.

## Methods

This cross-sectional study was approved by the University of Rochester’s institutional review board, which waived informed consent because data were anonymous and reviewed retrospectively. We followed the STROBE reporting guideline. An opioid reduction initiative was implemented in 2013 in our dental urgent care clinic.^5^ A multimodal analgesia strategy including gabapentin was initiated in 2020 to further minimize opioid use. We used prescription data from March 2021 to February 2022 to represent prescription patterns in 2022 and from 2012 to represent patterns before opioid reduction. The following information was retrieved from electronic health records: tooth extractions (routine or surgical), analgesics prescribed (single medication, opioid combinations, or multimodal analgesia including ≥2 medications [ibuprofen, acetaminophen, and gabapentin]), and follow-up visits for postoperative pain (patients returning for additional pain treatment were considered as having failed to achieve pain relief). More information is available in the eMethods in the Supplement.

Frequencies of different analgesic prescriptions after tooth extractions were compared between the 2 periods. Descriptive statistics, χ^2^ tests, and relative risks (RRs) with 95% CIs were used to assess changes in patterns of analgesic prescriptions and differences in failure rates. Data were analyzed with OpenEpi, version 3.1. Two-sided *P* < .05 was significant.

## Results

A total of 3357 patients (1715 [51.1%] female; mean age, 36 years [range, 18-93 years]) were prescribed analgesics after dental extractions in 2012 compared with 3785 (1941 [51.3%] male; mean age, 39 years [range, 18-93 years]) in 2022. The rate of nonopioid multimodal analgesia use was higher in 2022 (2367 patients [62.5%]) than in 2012 (7 [0.2%]). In all, 1166 patients (34.7%) received opioid combination analgesics after extractions in 2012. In 2022, no patients received opioids, but 1871 (49.4%) received acetaminophen/ibuprofen and 496 (13.1%) received gabapentin multimodal analgesia.

The Figure shows proportions of analgesics prescribed for dental extractions. No opioid prescriptions were recorded in 2022 after addition of gabapentin to the multimodal strategy.

**Figure.**
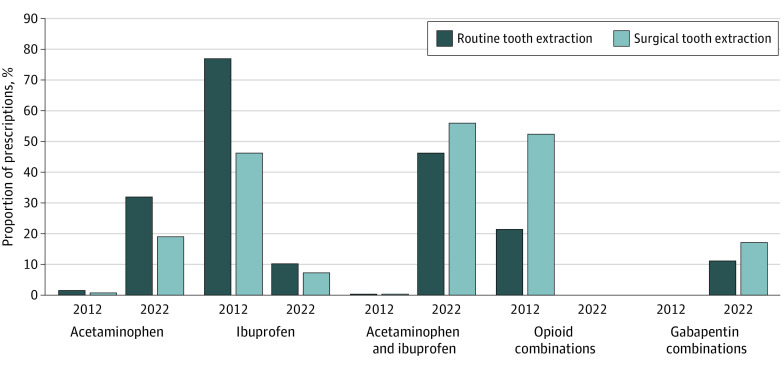
Proportions of Different Categories of Analgesics Prescribed After Dental Extractions During the 2 Study Periods Routine tooth extraction indicates removal of an erupted tooth or exposed tooth root without raising a soft-tissue flap or cutting and removing bone, and surgical tooth extraction indicates removal of an erupted or impacted tooth or buried tooth root that required cutting the soft tissue and cutting and removing the bone structure around the tooth.

Acetaminophen/ibuprofen had a failure rate (2.2%) significantly lower than that of gabapentin/acetaminophen or gabapentin/ibuprofen (4.4%; RR, 0.50; 95% CI, 0.31-0.83; *P* < .001) and that of opioids (21.4%; RR, 0.10; 95% CI, 0.08-0.14; *P* < .001). Failure rate for multimodal analgesia including gabapentin was significantly lower than for opioids (RR, 0.21; 95% CI, 0.14-0.31; *P* < .001) (Table).

**Table.** Pain Control Failure Rates After Routine and Surgical Tooth Extractions During the 2012 and 2022 Study Periods by Analgesic Category^a^

Analgesic	Routine extraction		Surgical extraction		Total	
	Patients, No.	Failure rate, No. (%)	Patients, No.	Failure rate, No. (%)	Patients, No.	Failure rate, No. (%)
Nonopioids						
Total	1493	22 (1.5)	881	42 (4.8)	2374	64 (2.7)
Acetaminophen/ibuprofen	1203	11 (0.9)	675	31 (4.6)	1878	42 (2.2)
Gabapentin combinations						
Total	290	11 (3.8)	206	11 (5.3)	496	22 (4.4)
Gabapentin/ibuprofen	57	3 (5.3)	44	3 (6.8)	101	6 (5.9)
Gabapentin/acetaminophen	233	8 (3.4)	162	8 (4.9)	395	16 (4.1)
Opioids						
Total	413	72 (17.4)	753	177 (23.5)	1166	249 (21.4)
Codeine/acetaminophen	98	9 (9.2)	122	10 (8.2)	220	19 (8.6)
Hydrocodone/acetaminophen	299	58 (19.4)	619	166 (26.8)	918	224 (24.4)
Other opioid combinations^b^	16	5 (31.3)	12	1 (8.3)	28	6 (21.4)

^a^ Failure rates are shown as the number (percentage) of patients who returned to the clinic for additional treatment of pain after receiving a tooth extraction and the prescribed analgesics. Routine tooth extraction indicates removal of an erupted tooth or exposed tooth root without raising a soft-tissue flap or cutting and removing bone, and surgical tooth extraction indicates removal of an erupted or impacted tooth or buried tooth root that required cutting the soft tissue and cutting and removing the bone structure around the tooth.

^b^ Included hydrocodone/ibuprofen and oxycodone/acetaminophen.

## Discussion

This study showed a shift in prescribing in our clinic from opioids and single-medication analgesics to nonopioids and multimodal analgesia to manage postoperative dental pain. Compared with 2012, when opioid combinations or ibuprofen alone were predominant, acetaminophen, ibuprofen, acetaminophen/ibuprofen, and gabapentin in a multimodal strategy were used more frequently in 2022.

No opioids were prescribed for dental pain from Mach 2021 to February 2022. Considering that approximately 1800 patients received more than 20 000 opioid pills annually in our clinic before implementation of the opioid reduction strategy,^5^ eliminating opioid prescriptions may mean that approximately 105 individuals annually will not develop new and persistent opioid use associated with treatment at our clinic.^1^

A Cochrane review demonstrated efficacy of gabapentin for acute dental pain.^6^ Gabapentin is not metabolized in the body and thus is safe in combination with other analgesics, such as acetaminophen or NSAIDs, providing a potential alternative to opioids, especially when acetaminophen/NSAIDs are contraindicated. A study limitation is that the number of patients returning for additional treatment is not an established metric for analgesic effectiveness. Clinical trials using validated outcome measures are needed to determine effectiveness and safety of this multimodal analgesia strategy.

## Notes

Supplement.
eMethods.
