# Supplementary material for: Comparison of Analgesic Prescriptions for Dental Pain and Patient Pain Outcomes Before vs After an Opioid Reduction Initiative
Source: JAMA Netw Open. 2022 Aug 17;5(8):e2227219. doi: 10.1001/jamanetworkopen.2022.27219 (PMC9386536; doi:10.1001/jamanetworkopen.2022.27219)
Supplement: Supplement. — eMethods. [file jamanetwopen-e2227219-s001.pdf]

Supplementary Online Content

Huang Q, Rasubala L, Gracely RH, Khan J, Eliav E, Ren Y. Comparison of analgesic prescriptions for dental pain and patient outcomes before vs after an opioid reduction initiative. *JAMA Netw Open*. 2022;5(8):e2227219. doi:10.1001/jamanetworkopen.2022.27219

eMethods.

This supplementary material has been provided by the authors to give readers additional information about their work.

© 2022 Huang Q et al. *JAMA Network Open*.

eMethods.

Objectives: The purpose of the present study was to compare patterns and effectiveness of analgesic prescriptions for acute dental pain before and after implementation of opioid reduction strategies in HDUC. Effectiveness was evaluated using a real-world measure of proportion of patients returning for additional pain treatment after receiving the prescribed analgesics. We hypothesized that combination analgesics with NSAIDs, acetaminophen and gabapentin as components minimize reliance on opioids by dentists for acute dental pain.

Study Population: All patient information were anonymized and de-identified prior to the analysis. A total of 8,955 and 14,765 patients visited the HDUC during the two 12-month periods in Year-2012 (January 2012 to December 2012) and Year-2022 (March 2021 to February 2022), respectively. Of these patients, 4,863 received dental extraction in Year-2012, compared to 7,056 in Year-2022. Among patients who received dental extractions, 3,357 (69.0%) were prescribed analgesics for pain in Year-2012, compared to 3,785 (53.6%) in Year-2022. The mean age of patients received dental extractions was 37 years (range 18 to 93) in Year-2012, and 39 years (range 18 to 97) in Year-2022. Sex of the patients were about equally distributed between female and male in both periods (51.6% female and 48.4% male in Year-2012, and 51.0% female and 49.0% male in Year-2022).

Definitions of routine or surgical extractions: Routine extraction was defined as removal of erupted tooth or exposed tooth root without raising a soft tissue flap or cutting and removing bone. Surgical extraction was defined as removal of erupted or impacted tooth or buried tooth root that required cutting the soft tissue, and cutting and removing bone structure around the tooth.

Definitions of multimodal analgesia vs. combination analgesics: Multimodal analgesia combines analgesics from two or more drug classes or analgesic techniques that employ different mechanisms of action, targeting different pain pathways in the peripheral and central nervous system, thus achieving a synergistic effect at lower analgesic doses [1-3]. Combination analgesics describe two medications compounded into the same tablet in this study, which included the opioids compound of hydrocodone plus acetaminophen, hydrocodone plus ibuprofen, codeine plus acetaminophen, and oxycodone plus acetaminophen.

Analgesic prescription guidelines: Opioid analgesics included hydrocodone, oxycodone, codeine in combination with acetaminophen and/or ibuprofen. Non-opioid analgesics included acetaminophen, NSAIDs (primarily ibuprofen), gabapentin and their combinations. Gabapentin was always used as a combination with either acetaminophen or ibuprofen. Guidelines for prescribing analgesics are as follows: In Year-2012, patients with mild pain were usually treated with over-the-counter medications including acetaminophen (325mg) and ibuprofen (200mg); those with moderate to severe pain were prescribed higher doses

© 2022 Huang Q et al. *JAMA Network Open*.

of ibuprofen (400-600mg) or opioid combinations, usually hydrocodone/acetaminophen 5/325mg, and patients expected to have severe pain were prescribed ibuprofen 600-800mg or opioid combinations such as hydrocodone/acetaminophen 7.5/325mg. In Year-2022, patients with mild pain were more likely prescribed with acetaminophen 500mg or ibuprofen 400mg; those with moderate to severe pain were prescribed ibuprofen 600mg or ibuprofen 400mg/acetaminophen 325mg combinations; and those expected to have severe pain were prescribed ibuprofen 400-600mg/acetaminophen 500mg combinations. For patients who had moderate to severe pain but could not take ibuprofen when indicated, they were usually prescribed opioid combinations in Year-2012, but were prescribed gabapentin 300mg/acetaminophen 500mg combinations in Year-2022 when indicated. Similarly, for patients who could not take acetaminophen when combination medications were indicated, hydrocodone/ibuprofen combinations were prescribed in Year-2012, but gabapentin 300mg/ibuprofen 400mg combinations were prescribed in Year-2022. All opioids and non-opioids were prescribed every 6 hours as needed for pain for 3 to 5 days except the gabapentin, which was prescribed every 12 hours as needed for pain.

Additional references for Method Supplement:

1. Chou R, Gordon DB, de Leon-Casasola OA, et al. Management of Postoperative Pain: A Clinical Practice Guideline From the American Pain Society, the American Society of Regional Anesthesia and Pain Medicine, and the American Society of Anesthesiologists' Committee on Regional Anesthesia, Executive Committee, and Administrative Council. *J Pain.* 2016;17(2):131-157.
2. Kehlet H, Dahl JB. The value of "multimodal" or "balanced analgesia" in postoperative pain treatment. *Anesth Analg.* 1993;77(5):1048-1056.
3. Polomano RC, Fillman M, Giordano NA, Vallerand AH, Nicely KLW, Jungquist CR. Multimodal Analgesia for Acute Postoperative and Trauma-Related Pain. *AJN The American Journal of Nursing.* 2017;117(3):S12-S26.

© 2022 Huang Q et al. *JAMA Network Open*.
